# A Solid-State Source of Single and Entangled Photons at Diamond SiV-Center Transitions Operating at 80K

**DOI:** 10.1021/acs.nanolett.3c01570

**Published:** 2023-06-28

**Authors:** Xin Cao, Jingzhong Yang, Tom Fandrich, Yiteng Zhang, Eddy P. Rugeramigabo, Benedikt Brechtken, Rolf J. Haug, Michael Zopf, Fei Ding

**Affiliations:** † Institut für Festkörperphysik, Leibniz Universität Hannover, Appelstraße 2, 30167, Hannover, Germany; ‡ Laboratorium für Nano- und Quantenengineering, Leibniz Universität Hannover, Schneiderberg 39, 30167, Hannover, Germany

**Keywords:** GaAs semiconductor quantum dots, single photons, entangled photon pairs, liquid nitrogen temperature, diamond color centers, SiV zero phonon line

## Abstract

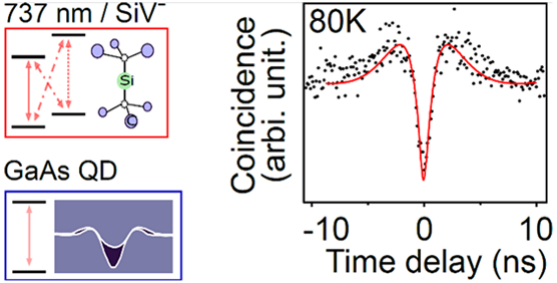

Large-scale quantum networks require the implementation of long-lived quantum memories as stationary nodes interacting with qubits of light. Epitaxially grown quantum dots hold great potential for the on-demand generation of single and entangled photons with high purity and indistinguishability. Coupling these emitters to memories with long coherence times enables the development of hybrid nanophotonic devices that incorporate the advantages of both systems. Here we report the first GaAs/AlGaAs quantum dots grown by the droplet etching and nanohole infilling method, emitting single photons with a narrow wavelength distribution (736.2 ± 1.7 nm) close to the zero-phonon line of silicon-vacancy centers. Polarization entangled photons are generated via the biexciton–exciton cascade with a fidelity of (0.73 ± 0.09). High single photon purity is maintained from 4 K (g^(2)^(0) = 0.07 ± 0.02) up to 80 K (g^(2)^(0) = 0.11 ± 0.01), therefore making this hybrid system technologically attractive for real-world quantum photonic applications.

Quantum repeaters are envisioned key components for large, distributed quantum networks.^1−3^ Their realization requires efficient single photon and entangled photon pair sources as well as quantum memory (QM) platforms to store quantum information imprinted on photons. On the one hand, semiconductor quantum dots (QDs) based on GaAs have proven to be excellent single and entangled photon sources in the last years: they are on-demand emitters,^4^ with ultrahigh single photon purity,^5^ entanglement fidelity,^6^ indistinguishability,^7^ and wavelength tunability.^8^ However, because QDs are embedded in a solid state matrix, the coherence time of the emitted photons is limited due to the coupling with the phonons in the matrix.^9^ On the other hand, QMs based on single atoms,^10^ atomic ensembles,^11^ and trapped ions^12^ exhibit long storage times up to milliseconds, even though the photon generation efficiency is low. Combining QDs with a QM platform allows for taking advantage of both systems by mapping quantum information encoded on photons to stationary network nodes with an adequate storage time. This would allow the basic implementation of quantum repeaters via the “node receives photon” protocol.^13^ Although several groups achieved wavelength matching between InAs, InGaAs, or GaAs QDs, on the one hand, and trapped Yb^+^ ions or Cs and Rb atomic vapor,^14−16^ on the other hand, such hybrid systems lack the compatibility with state-of-the-art semiconductor technology which limit a scalable application.

Defect centers in diamond are promising solid state platforms to store and read out single photons. The negatively charged silicon-vacancy (SiV) and nitrogen-vacancy (NV) centers have been widely studied.^17,18^ SiV offers an efficient light-matter interface together with long spin coherence times at cryogenic temperature (13 ms^19^). Single photon storage in SiV-centers efficiently coupled with nanocavities as well as coupling to ^13^C nuclear spins has been achieved, laying the foundations for implementation of practical quantum memory nodes.^20,21^ The SiV zero-phonon-line (ZPL) is spectrally located at about 737 nm,^22^ within the emission regime of GaAs QDs. However, coupling experiments between SiV-centers and GaAs QDs have not yet been reported yet. To achieve this, the emission wavelength must match the ZPL. One possibility is to grow GaAs QDs via droplet epitaxy^23^ with the emission wavelength tuning controlled by the metal droplet size. Alternatively, *in situ* local droplet etching (LDE) can be^24,25^ used, in which case the QD emission wavelength is optimized by varying the amount of GaAs infilled in the etched nanoholes.^26,27^

In this work, we present novel GaAs QDs with emission wavelengths at the SiV-ZPL and investigate their spectral properties and single photon characteristics up to temperatures of 80 K. The QD samples are grown via molecular beam epitaxy by means of *in situ* LDE of nanoholes and subsequent infilling.^28^ This technique allows for strain-free lattice matched growth of highly symmetric QDs that have typically been optimized for emission at 780 or 795 nm^29,30^ to match rubidium atomic transitions. As displayed in Figure 1a, an Al_0.3_Ga_0.7_As barrier is first grown on top of the GaAs buffer layer. Thereafter, Al droplets are deposited to etch nanoholes on the Al_0.3_Ga_0.7_As surface. Figure 1b and c show an atomic force microscope image and a line profile of a typical nanohole, respectively. The line profile reveals nanoholes with a depth of about 11 nm and a diameter of 100 nm. After nanohole infilling with GaAs, a top Al_0.3_Ga_0.7_As barrier layer is grown, followed by a GaAs cap layer.

**Figure 1:**
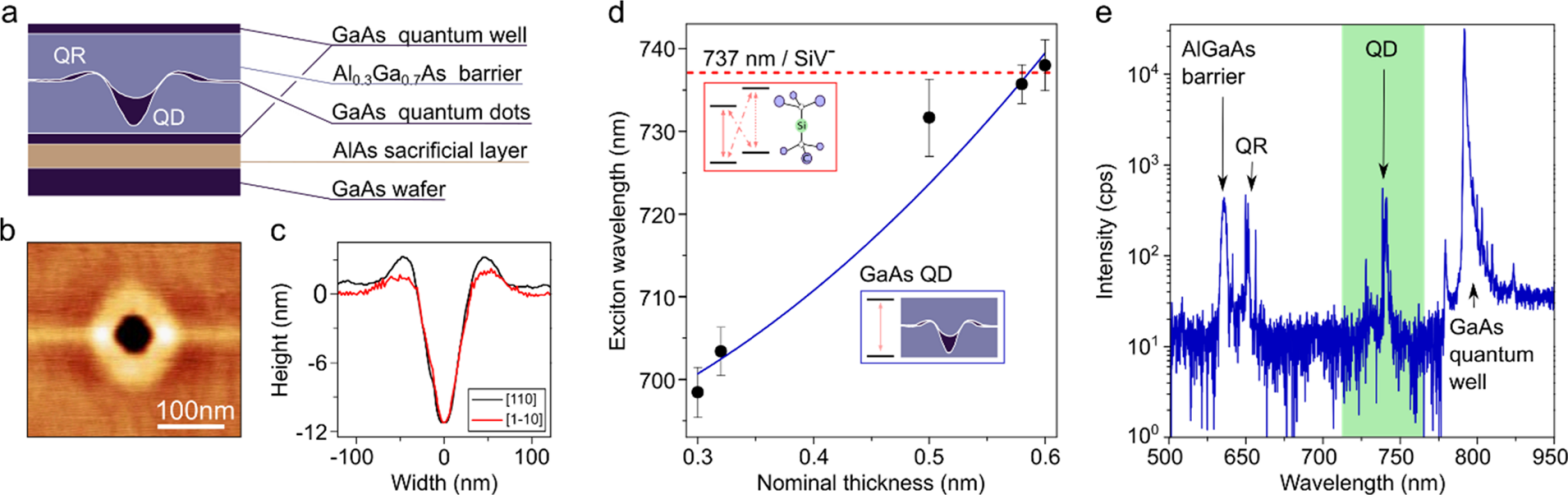
(a) Sketch of the sample structure (not to scale) with a quantum dot (QD) and a quantum ring (QR). (b) Atomic force microscopy image of an Al etched nanohole on an Al_0.3_Ga_0.7_As surface and (c) the corresponding height profile along the [110] and [1–10] directions. (d) Neutral exciton emission wavelength of the GaAs QDs as a function of deposited GaAs nominal thickness. The points are experimental data, the solid blue line is a fit, and the dotted red line shows the SiV-ZPL emission. (e) Micro-PL spectrum of the wafer at the location of a representative single QD emitting at around 737 nm, measured at a temperature of *T* = 4 K.

Due to the nanohole dimensions, the energy of the confined charges and excitonic complexes is mostly determined by the confinement in the growth direction and thus the amount of in-filled GaAs. The QD emission energy is approximated by , where m* is the exciton effective mass and *d*_z_ is the thickness of GaAs in the nanohole. Therefore, adjusting the deposited amount of GaAs allows for fine-tuning the QD emission wavelength, as shown in Figure 1d. Wavelength optimization is accompanied by microphotoluminescence measurements performed at *T* = 4 K. As expected, reducing the nominal thickness of infilled GaAs leads to blue-shifting of the emission wavelength of the GaAs QDs. A typical wide-range spectrum is presented in Figure 1e. In addition to emissions from the GaAs quantum well and AlGaAs barrier, two types of localized and spectrally well-defined emission peaks are observed at different wavelengths. The peaks highlighted in green correspond to the emission from QDs (material filled inside the nanohole), while the blue-shifted emission close to the AlGaAs barrier emission is attributed to the residual material around nanohole borders, forming a quantum ring (QR).^31^ QR formation can be explained by the LDE process, which results in the formation of a ring-shaped structure around the nanohole (see Figure 1b, c) because of the crystallization of residual droplet material. During nanohole infilling, most of the GaAs migrates inside the nanohole to minimize surface energy, with some GaAs material accumulating around the outer ring structure.

QD emission wavelengths can be deterministically tuned toward the SiV-center ZPL by optimizing growth parameters. QRs can also be tuned to emit, e.g., at the NV-center ZPL, however less deterministically. Therefore, QDs are particularly attractive and will be the focus here. We find that the QDs will emit around the ZPL of SiV-centers in diamond with a GaAs nominal thickness of 0.53–0.6 nm.

Individual GaAs QDs are optically excited at *T* = 4 K by 532 nm continuous wave laser light focused by an objective with a 100× magnification and 0.7 numerical aperture. The PL spectrum of a representative QD matching the SiV-center ZPL is presented in Figure 2a. The spectrum is similar to that of GaAs QDs emitting at 780–795 nm, with a dominant neutral exciton (X) emission and several red-shifted transitions typically containing the neutral biexciton (XX) and several hot trion emissions.^32^ For better scalability and optimal coupling between QDs and SiV-centers, it is desirable that most of the QDs on the same wafer can match the ZPL, which can be ensured by the homogeneous infilling of GaAs into the nanoholes. The left inset of Figure 2a shows the distribution of X wavelengths across the wafer, based on measurements from 32 dots and a nominal GaAs thickness of 0.56 nm. The resulting wavelength distribution is 736.2 ± 1.7 nm, matching well with the SiV-ZPL. We identified the XX peak by measuring its linear polarization. The XX emission reveals the same fine structure splitting (FSS) as the X emission (see supplementary). The coherence of the emitted X photons is measured using a Michelson interferometer and displayed in the right inset of Figure 2a, showing the interference visibility over the delay time difference between the two interferometer arms. A coherence time of 115.3 ± 5.5 ps for the X photons is extracted using a model that considers a spectral line with Gaussian broadening. This value is below the radiative lifetime-limit and points toward the presence of fast spectral diffusion due to charge traps in the QD vicinity.^33^

**Figure 2:**
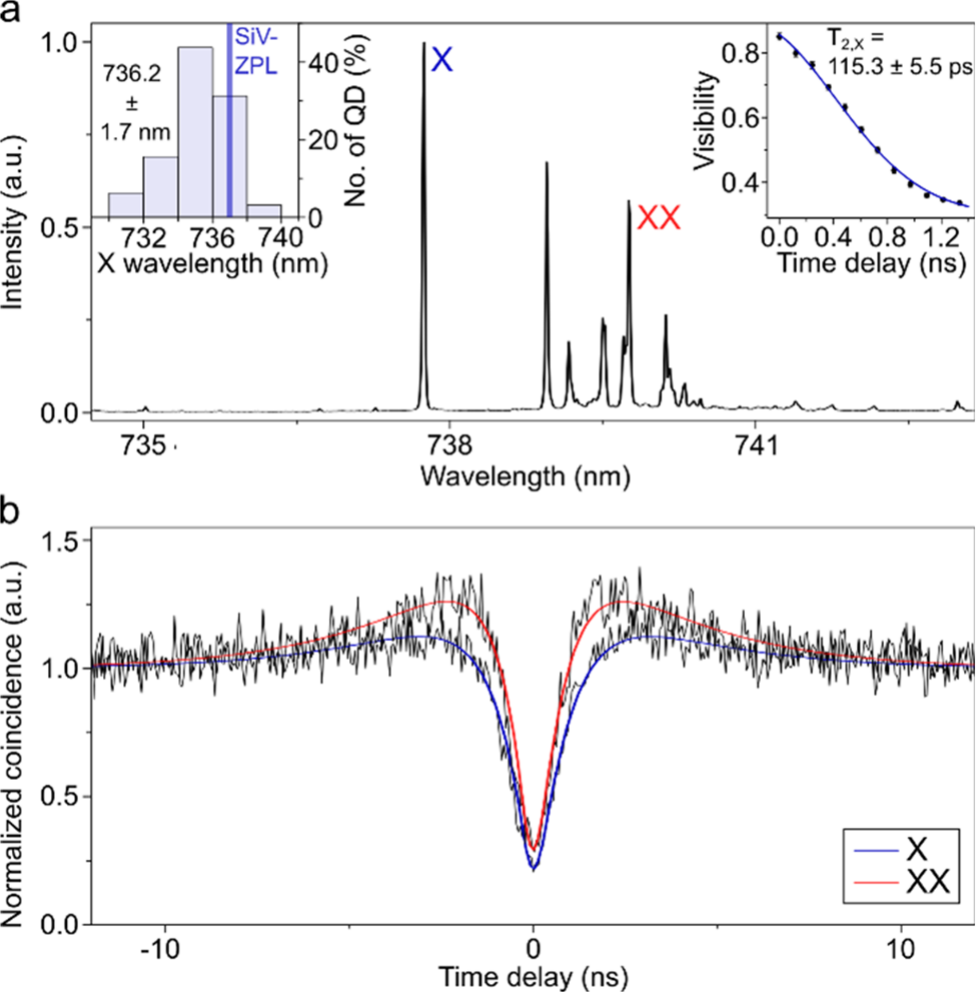
(a) PL spectrum of a representative GaAs QD with the neutral exciton wavelength matching the SiV-ZPL. X and XX represent exciton and biexciton peaks, respectively. Left inset: X emission wavelength distribution was obtained by measuring 32 quantum dots. Right inset: Interference visibility of the X emission over the time delay in an unbalanced Michelson interferometer. A Gaussian line shape is revealed, and a coherence time of *T*_2, X_ = 115.3 ± 5.5 ps is determined. (b) Second-order autocorrelation measurements of X and XX emissions, respectively. The solid lines denote the theoretical model.

High single photon purity is an important property for quantum communication, e.g., to avoid photon number splitting attacks in quantum cryptography protocols.^34^ We performed second-order autocorrelation measurements with a standard Hanbury Brown and Twiss setup while exciting the QDs off-resonantly with a continuous wave laser. The photons are detected by avalanche photodiodes with a time resolution of 350 ps. The normalized coincidences are shown in Figure 2b together with the following theoretical model of the delay time dependent autocorrelation function:^16,35,36^
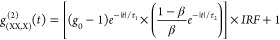
with *g*_0_ the value of single photon purity, τ_1_ the time resulting from the pumping and decay process, β the fraction of time when the QD is in an “on” state, τ_2_ the time of the blinking process and the instrument response function *IRF.* Values of *g*_XX_^(2)^ (0) and *g*_X_^(2)^ (0) of 0.08 ± 0.01 and 0.07 ± 0.01 for X and XX are obtained, respectively, confirming a strong single photon character of the emission. The bunching effect is slightly stronger for the XX transition compared with the X transition. Because the XX peak is in spectral proximity to other charged exciton emissions (see Figure 2a), a small fraction from these transitions might also be detected during the measurement due to imperfect spectral filtering, leading to the lower probability of forming two electron–hole pairs under off-resonant excitation.^37,38^

Polarization entanglement of multiphoton states is an important resource for quantum communication applications. The polarization state of photons emitted by a QD can be mapped to the spin state in a SiV-center. If one photon from an entangled pair is used for that, polarization-spin entanglement can be achieved between the flying qubit (remaining photon) and stationary qubit (spin state in SiV-center). Together with entanglement swapping, various such systems can be used as building blocks for quantum repeaters to overcome the current distance limits of quantum communication.^13^ Therefore, we now look at the optical properties that are relevant for generating polarization-entangled photons by exploiting the XX-X cascade. We perform a statistical measurement of the distribution of exciton FSS in the sample which results in a value of 7.0 ± 4.6 μeV (see supplementary). Al droplet etching has been shown to yield symmetric nanoholes on AlGaAs surfaces, which eventually result in low FSS values in QDs and therefore enable the emission of polarization-entangled XX and X photons without the need of postgrowth tuning or temporal photon selection.^25,29^

A QD with an FSS of 4.9 μeV is chosen for entanglement fidelity measurement. Instead of a maximally entangled Bell state, the finite FSS leads to the emission of a Bell state with a phase factor that depends on the respective exciton decay time resulting in an oscillating entanglement fidelity to one particular Bell state.^39^ Six polarization-resolved second-order cross-correlation functions are obtained and shown in Figure 3 for the rectilinear (H/V), diagonal (D/A) and circular (R/L) polarization bases together with the employed model (see supplementary). Because of the angular momentum conservation, antibunching and bunching should ideally be observed in the V-V, D-D, and R-L bases; however, for the V-H, D-A, and R-R bases, only antibunching is expected. Our results differ from this expectation for the following reasons: First, because of the additional phase in the entanglement due to the FSS and t_1_, we expect oscillations in the D-D/D-A and R-R/R-L bases. Since the oscillation period is of the same order of magnitude as the time resolution of the employed avalanche photodiodes, quantum beat effects are washed out. The second reason is a shift between the QD symmetry axes and the axes of the measurement system. By simultaneously modeling all six data sets, an entanglement fidelity of 0.73 ± 0.09 and an exciton lifetime of (368 ± 21) ps are obtained. The oscillation in the two-photon polarization states can be seen in the unconvoluted model in Figure 3, in particular in the RR and RL bases.

**Figure 3:**
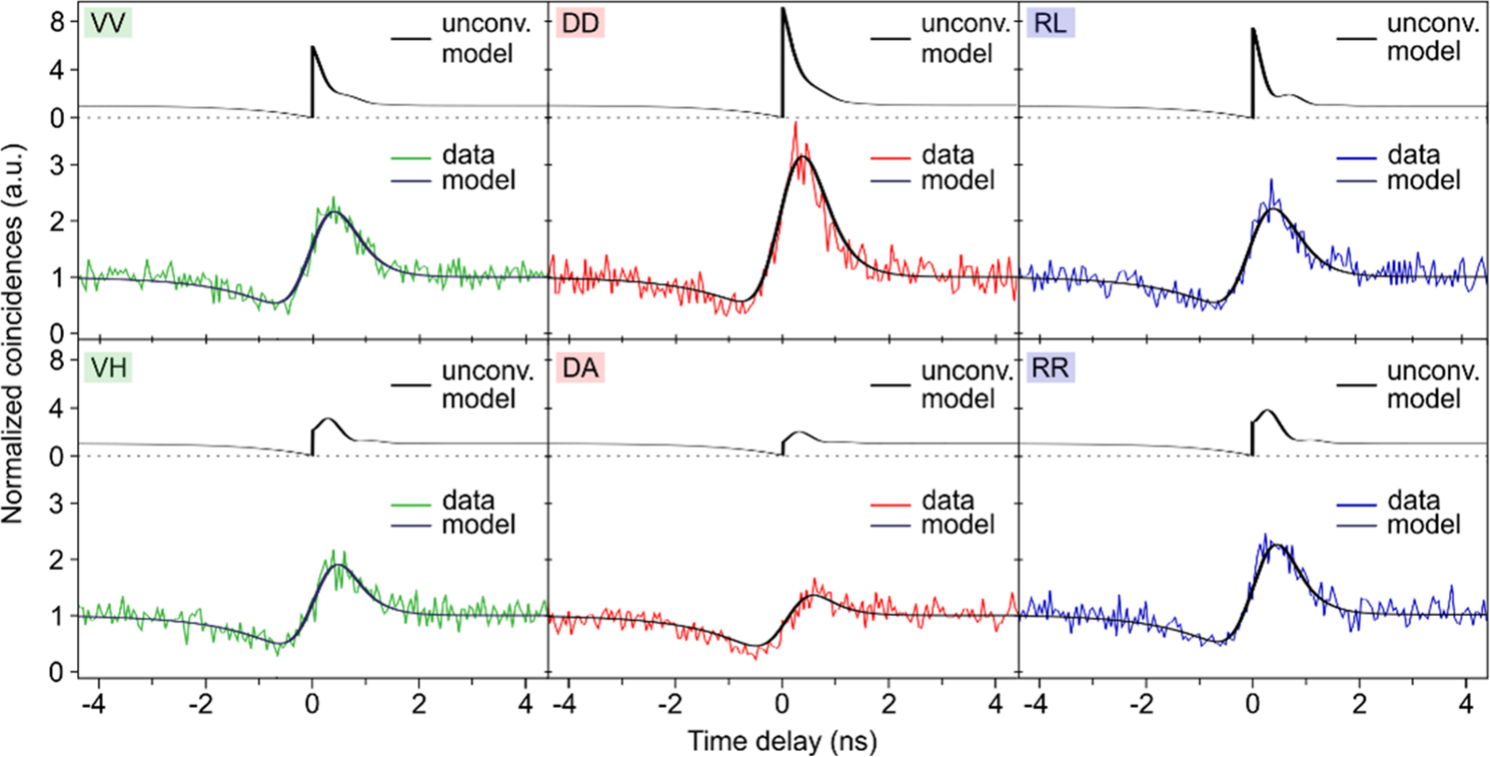
Polarization-resolved cross-correlation measurements of the XX-X emission from the biexciton–exciton cascade with polarization projection onto the H-V, D-A, and R-L bases, respectively. The solid line represents the theoretical model, revealing an entanglement fidelity to the Bell state Φ^+^ of (0.73 ± 0.09).

The observed X lifetime is slightly longer than what is typically observed for GaAs QDs emitting at 780 nm,^29^ which may be attributed to the stronger confinement due to the smaller size of the QDs here. However, the lifetime is still in a range that is expected for a system with weak carrier confinement.^40,41^ Higher entanglement fidelities may be achieved by reducing the X lifetime via Purcell enhancement in photonic nanostructures with embedded QDs. Also, postgrowth tuning techniques such as anisotropic strain-tuning can reduce the FSS. Further improvements are expected when utilizing pulsed two-photon resonant excitation of the XX state and single photon detectors with better time resolution at this wavelength.

The measurements conducted so far are all performed at 4 K. Up to now, epitaxial QDs were reported with single photon emission at room temperature or even higher temperature in material systems with a larger energy gap.^42−44^ At liquid nitrogen temperatures, strained QDs have been observed to emit single photons at telecommunication wavelengths.^45,46^ Strain-free GaAs/AlGaAs QDs have not yet been reported to emit single photons in the visible wavelength range at the liquid nitrogen temperature. Figure 4a shows the PL spectra of a representative QD at different temperatures. With increasing temperature, the emission peaks are red-shifted, because of the shrinking band gap. Above 40 K phonon scattering becomes more prominent, featuring an obvious phonon wing. Nevertheless, the broadened neutral X peak is still clearly visible at 80 K. We attribute this effect to stronger quantum confinement, leading to an increased electron–hole interaction and level spacing. The integrated intensity of the neutral X peak is displayed as a function of inverse temperature in Figure 4b (solid dots and green curve). The Arrhenius function is used as a model to the data:
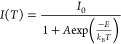
where *I*_0_ is the initial intensity, *A* is the coupling constant, *k*_B_ is the Boltzmann constant, and *E* is the activation energy for nonradiative decay. We extract an activation energy of 16.7 ± 1.5 meV. In GaAs QDs emitting at around 785 nm, an energy difference of 13 meV between 1e^1^ and 2e^1^ shells (ground and first excited electron state in the conduction band) is reported.^40^ We presume that the increased spacing of energy eigenstates is directly related to a decrease in QD size. With sufficient thermal energy, the charge carrier can escape the ground state and occupy higher excited states or, at higher temperatures, even reach the energy bands of the barrier material, resulting in PL intensity quenching of the neutral X.^47^

**Figure 4:**
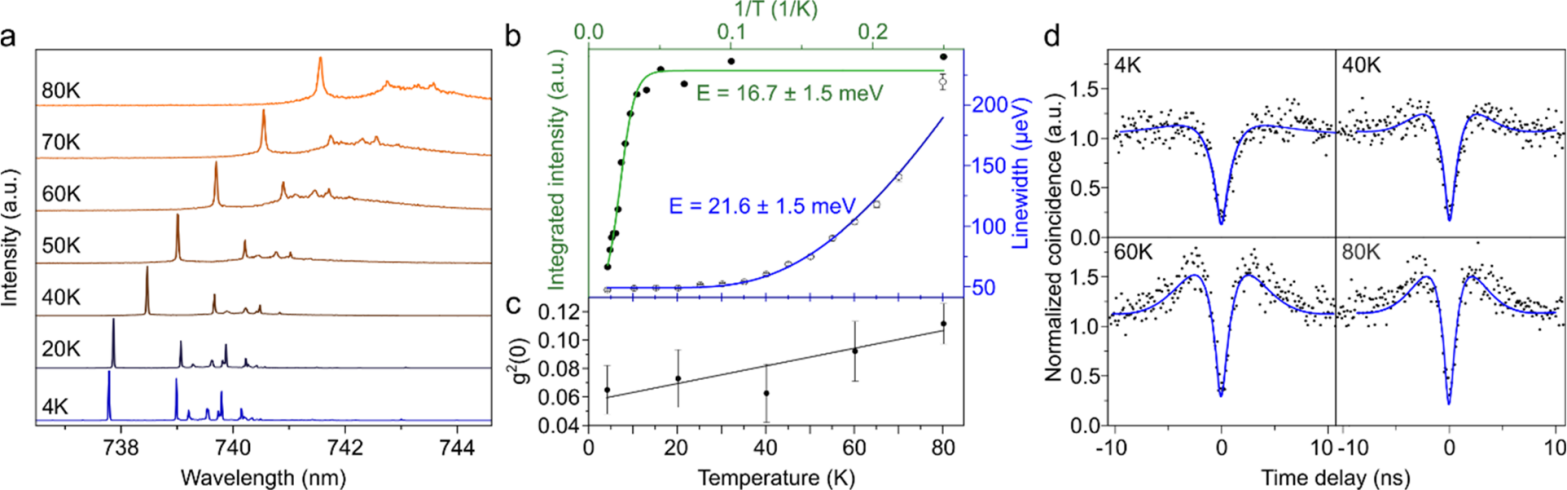
(a) Temperature-dependent PL spectra showing a red-shift and emission broadening toward higher temperatures. The X emission is clearly observable even at liquid N_2_ temperatures. (b) Integrated X peak intensity as a function of inverse temperature (filled circles and green curve) and X line width as a function of temperature (open circles and blue curve). (c) Values of g^(2)^(0) over different temperatures. (d) Second-order autocorrelation measurements of X emission at different temperatures (4, 40, 60, and 80 K).

The line width after subtraction of the acoustic phonon background of the neutral X peak at different temperatures is presented in Figure 4b (open circles and blue curve). The line width remains almost constant below 40 K (Spectrometer resolution limit 38 μeV) and increases with ∼1/(exp(1/*T*)) at higher temperatures. This can be modeled by an activated behavior as follows:
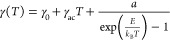
where γ(*T*) is the line width at a temperature *T*, γ_0_ is the line width at *T* = 0 K, γ_ac_ stands for acoustic phonon broadening, *a* is a constant, *k*_B_ is the Boltzmann constant, and E is the energy for optical phonon broadening.^48^ The extracted energy of the longitudinal optical phonons coupling to the exciton is 21.6 ± 1.5 meV.

High single photon purity is a prerequisite for practical applications at a liquid nitrogen temperature. Since the X peak is still clearly visible at 80 K, we perform second-order autocorrelation measurements from 4 K over 40, 60, and up to 80 K, respectively. As shown in Figure 4c, the g^(2)^(0) value does not change significantly between 4 and 40 K, and then slightly increases. At 80 K, there is still pure single photon emission with g^(2)^(0) of about 0.1. With increasing temperature, the antibunching dip at *T* = 0 is getting narrower. This is because, on the one hand, the effective pumping rate is higher at high temperatures.^49,50^ On the other hand, charge carriers can relax more easily between different energy states in the QD, since the phonon bottleneck is overcome easier at higher temperature.^51^ At 60 and 80 K, the bunching effect in the vicinity of zero time delay is more prominent. This may be explained by the tunneling of charge carriers in and out of impurity induced trap states nearby the QD.^36^ At high temperatures, such processes may become more pronounced due to the increased thermal energy.

In conclusion, we have grown GaAs QDs and finely adjusted the infilling amount to match the ZPL of the SiV-centers in diamond. Nanoholes of homogeneous depth were formed during Al droplet etching, which together with the deposition of nominally 0.56 nm GaAs results in a narrow wavelength distribution of (736.2 ± 1.7) nm. Both neutral X and XX peaks are clearly distinguished by measuring either their linear polarization state or their second-order cross-correlation functions. Both X and XX emit pure single photons, which is a prerequisite for quantum information processing. The entanglement fidelity above the classical limit of 0.5 shows that these GaAs QDs are polarization-entangled light sources with a strong potential for coupling to SiV-center-based quantum memories. The slightly longer observed X lifetime compared with GaAs QDs emitting at 780 nm can prove beneficial for coupling to SiV- centers in diamond, which typically exhibit decay times of 1.7 ns.^52^ Given that Fourier-transform limited line widths can be reached in the QD system, the bandwidths would have a reasonably small mismatch (ratio of bandwidths of <5). By slightly modifying the photonic density of states around the quantum dot using photonic structures, an efficient coupling can therefore be envisioned. A further advantage of the presented QD based single photon emitter is the operability above liquid nitrogen temperature, enhancing its practical feasibility.

## Supporting Information Available

Information about polarization dependent photoluminescence measurements and statistical measurements on the exciton fine structure; detailed explanations about the employed model for the polarization-resolved cross-correlation measurements (PDF)
