# Supplementary material for: A Solid-State Source of Single and Entangled Photons at Diamond SiV-Center Transitions Operating at 80K
Source: Nano Lett. 2023 Jun 28;23(13):6109–15. doi: 10.1021/acs.nanolett.3c01570 (PMC10347697; doi:10.1021/acs.nanolett.3c01570)
Supplement: Supplementary file 1 — nl3c01570_si_001.pdf [file nl3c01570_si_001.pdf]

Supplemental material

A solid-state source of single and entangled photons at diamond SiV-center transitions operating at 80K

Xin Cao[1], Jingzhong Yang[1], Tom Fandrich[1], Yiteng Zhang[1], Eddy P. Rugeramigabo[1], Benedikt Brechtken[1], Rolf J. Haug[1,2], Michael Zopf[1]□, Fei Ding[1,2]□

1. Institut für Festkörperphysik, Leibniz Universität Hannover, Appelstraße 2, 30167, Hannover, Germany

2. Laboratorium für Nano- und Quantenengineering, Leibniz Universität Hannover, Schneiderberg 39, 30167, Hannover, Germany

Email: michael.zopf@fkp.uni-hannover.de

Figure S1 a illustrates the emission energy over the linear polarization angle of the X and XX emission of the QD used for the polarization-resolved cross-correlation measurements in Figure 3. The oscillations with a phase shift of π reveal an exciton fine structure splitting (FSS) of 3.3 ± 0.2 μeV A statistical measurement comprising 39 dots is displayed in Figure S1 b and results in an average detected FSS value of 7.0 ± 4.6 μeV.

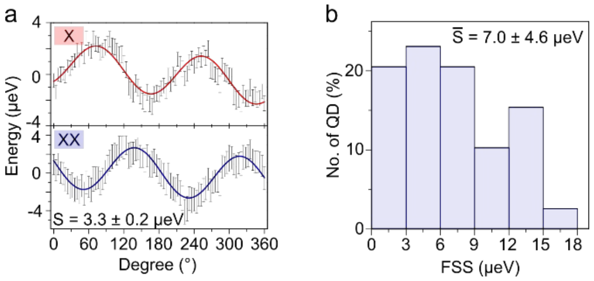

Figure S1. (a) Polarization-resolved emission energy of the X and XX transitions shown in figure 2 (a), revealing a finite exciton fine structure splitting. (b) Statistical distribution of neutral exciton fine structure based on 39 quantum dots.

Entangled-photon pairs can be generated via the biexciton-exciton radiative cascade. The polarization state of this decay is described by

$$|\Psi(t)\rangle = \frac{1}{\sqrt{2}}\left(|H_{XX}H_X\rangle + e^{-\frac{i}{\hbar}St_1}|V_{XX}V_X\rangle\right)\#$$

where S is the FSS amplitude between both bright exciton states, t_1 is the time the QD stays in the exciton state and $|H\rangle, |V\rangle$ are the horizontal (H) and vertical (V) polarization states. For GaAs QDs, a maximally entangled Bell state Φ^+ should be in principle measured if the fine structure splitting is 0. In our case, the FSS of the chosen QD is S = 4.9 μeV, with a time-averaged exciton lifetime of $T_1 = (368 \pm 21)$ ps(extracted using the model below). The non-vanishing FSS and the determined lifetime lead to the emission of a Bell state with a phase factor that depends on the respective exciton decay time, resulting in an entanglement fidelity that oscillates with the detection delay between XX and X photons[1]. Six polarization-resolved second-order cross-correlation functions are obtained and shown in Figure 3 for the rectilinear (H/V), diagonal (D/A) and circular (R/L) polarization bases. In the V-V polarization base, when the detection of the XX triggers as the 'Start' in the time correlated single photon counting system, the XX will not be re-excited until the X recombines via photon emission. Therefore, an anti-bunching dip occurs at zero-time delay, and a bunching shoulder appears afterwards. In the V-H base, when the polarization of XX is V, in principle, X should also be V polarized. In the H polarization base, the X photons will not be detected by the detector, only an anti-bunching dip should be observed. Because of the angular momentum conservation, anti-bunching and bunching should be observed in V-V, D-D and R-L base; while for V-H, D-A and R-R base, only anti-bunching is expected. However, our results differ from this theoretical expectation as shown in Fig. 3. Because of the additional phase in the entanglement due to FSS, we expect oscillations in the D-D/D-A and R-R/R-L bases. Additionally, since the oscillation period is in the same order of magnitude like the time resolution of the employed APDs of the quantum dot, quantum beat effects are washed out. The second difference to the expected behavior is the existing small bunching in the D-A base and large bunching in the V-H base. The main reason is the misalignment of the linear polarization states between the quantum dot defined by its symmetry and the respective detection bases.

The shift between the QD symmetry axes and the axes of the measurement system is implemented in the model in the following way: It is possible define any arbitrary polarization state on a point at the surface of the Poincaré sphere with two angles θ and ϕ. The horizontal and vertical polarizations constitute the north and south pole of the sphere respectively[2]. It follows:

$$|P(\theta,\phi)\rangle = \cos\left(\theta/2\right)|H\rangle + e^{i\phi}\sin\left(\theta/2\right)|V\rangle.\#$$

The values for the angles θ and ϕ of the six polarization states can be found in TABLE I. To include the shift θ_S, we need to apply the rotational operator [3]

$$R(\theta_S) = \begin{bmatrix} \cos\left(\theta_S/2\right) & -\sin\left(\theta_S/2\right) \\ \sin\left(\theta_S/2\right) & \cos\left(\theta_S/2\right) \end{bmatrix}\#$$

to the polarization state $|P(\theta,\phi)\rangle$. Assuming the biexciton with polarization P_1 was detected at $t = 0$, the probability to detect the cascaded exciton with polarization P_2 at the time *t*, is given by[1]:

$$p\left[t,P'_1(\theta_1,\phi_1,\theta_S),P'_2(\theta_2,\phi_2,\theta_S)\right] = \frac{e^{-\frac{t}{T_1}}}{T_1}\langle P'_1,P'_2|\rho(t,f)|P'_1,P'_2\rangle \# \\ \propto g_b^{(2)}(t,f,\theta_S,P'_1,P'_2)$$

where T_1 is the lifetime of the exciton and $P'(\theta,\phi,\theta_S) = R(\theta_S)P(\theta,\phi)$. The first factor stems from the Poissonian decay probability when the quantum dot is in the excited state. This probability rate is proportional to the bunching of the biexciton-exciton cascade. The exact time-dependent bunching factors for the six polarization combinations are shown in TABLE I.

Eventually, the fidelity can be obtained by modelling the datasets of the second order cross-correlation functions:

$$g_{i,j}^{(2)}(t,f) = \left[\Theta(-t)(g_0 - 1)e^{-\frac{|t|}{T_{d,1}}} + \Theta(t)(g_0 - 1)e^{-\frac{|t|}{T_{d,2}}} + \Theta(t)g_b^{(2)}(t,f,\theta_S,i,j)\right] * IRF + 1\#$$

where i,j is polarization state, $\Theta(t)$ the Heaviside step function, g_0 the anti-bunching parameter and $T_{d,1}(T_{d,2})$ the decay time of the anti-bunching. The factor $g_b^{(2)}(t,f,\theta_S,i,j)$ describes the bunching of the time-resolved biexciton-exciton cascade in dependence of the fidelity *f* and the misalignment or shift θ_S between quantum dot eigenbase and detection basis (see discussion above). Additionally, this model is convoluted with the impulse response function *IRF* of the APD detectors to include the temporal response of the system.

To model $g_b^{(2)}(t,f,\theta_S,i,j)$ and thus the fidelity, we assume that the entangled-photon pair polarization matrix ρ is described by the mixed state $\rho = \alpha\,\rho_{pure}(t) + (1-\alpha)\rho_{class}$, where $\rho_{pure}(t) = |\Psi(t)\rangle\langle\Psi(t)|$ is the pure two-photon density matrix and $\rho_{class} = \frac{1}{2}\,diag(1,0,0,1)$ the density matrix of classical correlated photons. The factor α describes the proportion of the pure state in the mixed state. For $\alpha = 1$ (0), we derive a pure (classical) density matrix. Rewriting this equation in terms of fidelity, we acquire $\alpha = (2f-1)$ with $0.5 \leq f \leq 1$ and thus

$$\rho = (2f-1)\,\rho_{pure}(t) + 2(1-f)\rho_{class}.\#$$

By simultaneously modelling all six datasets, an entanglement fidelity of 0.73 ± 0.09, a shift of (2.24 ± 0.14) rad and exciton lifetime (368 ± 21) ps are obtained. Due to the time resolution of 360 ps of the two APDs and the large FSS of the quantum dot, the two-photon polarization state oscillates, as can be seen in the unconvoluted model in Fig. 3 in particular in the RR and RL bases.

Table I. Time-resolved bunching factor in dependence of the six polarization combinations

Polarization biexciton $\lvert P_1(\theta,\phi)\rangle$	Polarization exciton $\lvert P_2(\theta,\phi)\rangle$	Time-resolved bunching factor $g_b^{(2)}(t,f,\theta_S,P'_1,P'_2) \Big/ \left(g_{0,b}\, e^{-\frac{t}{T_1}}\right)$
$\lvert V\rangle = \lvert P(\pi,\phi)\rangle$	$\lvert V\rangle = \lvert P(\pi,\phi)\rangle$	$\frac{1}{4}\left[1 + cos^2(\theta_S) + (2f-1)sin^2(\theta_S)\cos(St/\hbar)\right]$
	$\lvert H\rangle = \lvert P(0,\phi)\rangle$	$\frac{1}{4}\left[1 - cos^2(\theta_S) - (2f-1)sin^2(\theta_S)\cos(St/\hbar)\right]$
$\lvert D\rangle = \lvert P\left(\frac{\pi}{2},0\right)\rangle$	$\lvert D\rangle = \lvert P\left(\frac{\pi}{2},0\right)\rangle$	$\frac{1}{4}\left[1 + sin^2(\theta_S) + (2f-1)cos^2(\theta_S)\cos(St/\hbar)\right]$
	$\lvert A\rangle = \lvert P\left(\frac{\pi}{2},\pi\right)\rangle$	$\frac{1}{4}\left[1 - sin^2(\theta_S) - (2f-1)sin^2(\theta_S)\cos(St/\hbar)\right]$
$\lvert R\rangle = \lvert P\left(\frac{\pi}{2},\frac{3\pi}{2}\right)\rangle$	$\lvert L\rangle = \lvert P\left(\frac{\pi}{2},\frac{\pi}{2}\right)\rangle$	$\frac{1}{4}\left[1 + (2f-1)\cos(St/\hbar)\right]$
	$\lvert R\rangle = \lvert P\left(\frac{\pi}{2},\frac{3\pi}{2}\right)\rangle$	$\frac{1}{4}\left[1 - (2f-1)\cos(St/\hbar)\right]$

[1] Winik, R. et al. On-demand source of maximally entangled photon pairs using the biexciton-exciton radiative cascade. Phys. Rev. B 95, 235435 (2017).

[2] Scully, M. O. & Zubairy, M. S. Quantum Optics. (Cambridge University Press, 1997).

[3] Nielsen, M. A. & Chuang, I. L. Quantum computation and quantum information. (Cambridge University Press, 2010).
